# Clinicopathological Significance of *CDKN2A* Promoter Hypermethylation Frequency with Pancreatic Cancer

**DOI:** 10.1038/srep13563

**Published:** 2015-09-04

**Authors:** Bo Tang, Yang Li, Guangying Qi, Shengguang Yuan, Zhenran Wang, Shuiping Yu, Bo Li, Songqing He

**Affiliations:** 1 Laboratory of Liver Injury and Repair Molecular Medicine, Guilin Medical University, Guilin, 541001, Guangxi, People’s Republic of China; 2 Department of Hepatobiliary Surgery, Guilin Medical University, Affiliated Hospital, Guilin, 541001, Guangxi, People’s Republic of China; 3 Department of Medical Oncology, Guilin Medical University, Affiliated Hospital, Guilin, 541001, Guangxi, People’s Republic of China; 4 Department of Pathology and Physiopathology, Guilin Medical University, Guilin, Guangxi 541004, China

## Abstract

The prognosis of pancreatic cancer patients is very poor, with a 5-year survival of less than 6%. Previous studies demonstrated that the loss of function of CDKN2A is mainly caused by the hypermethylation of *CDKN2A* gene promoter; however, whether or not it is associated with the incidence of pancreatic cancer still remains unclear. In this study, we systematically reviewed the association between *CDKN2A* promoter methylation and pancreatic cancer using meta-analysis methods. The pooled data were analyzed by Review Manager 5.2. Fourteen studies eligible studies, including 418 pancreatic cancer, 155 pancreatic intraepithelial neoplasia (PanINs) and 45 chronic pancreatitis (CP) patients were analyzed. We observed that the frequency of *CDKN2A* methylation was significantly higher in pancreatic cancer patients than in normal healthy controls, the pooled OR = 17.19, 95% CI = 8.72–33.86, P < 0.00001. The frequency of *CDKN2A* methylation was also significantly higher in PanINs patients than that in normal individual controls, OR = 12.35, 95% CI = 1.70–89.89, P = 0.01. In addition, *CDKN2A* methylation was associated with worse survival in pancreatic cancer, HR = 4.46, 95% CI = 1.37–14.53, P = 0.01. The results strongly suggest that *CDKN2A* methylation is correlated with an increased risk of pancreatic cancer. *CDKN2A* methylation plays a critical role in pancreatic carcinogenesis and may serve as a prognostic marker.

Pancreatic cancer has a high mortality rate and is the 7^th^ most frequent cause of cancer death^1^. It is estimated that 43,920 people in the United States have suffered from pancreatic cancer, and 37,390 people died from pancreatic cancer in 2012^2^. Pancreatic cancer is a devastating disease with poor survival at advanced stages. Over the last two decades, the 5-year overall survival for pancreatic cancer only slightly improved despite the death rates of most cancers have decreased due to improvements in early diagnosis and efficient treatments^3^ .The insidious onset, lack of effective screening and early biomarker detection methods, as well as few efficient therapies (due to the complicated cellular and molecular makeup of the pancreatic tumors and their surrounding microenvironment) contribute to the unsatisfied clinical outcomes^4^. Therefore, biomarkers for early diagnosis and new/effective treatments are urgently needed.

In exocrine origin, pancreatic malignancy display heterogeneous glandular and duct-like structure with infiltration of the most pancreatic parenchyma and partial desmoplastic stroma. This typical pancreatic ductal adenocarcinoma (PDA) is a highly invasive metastatic disease evolved from a premalignant lesion^5^. Although PDA shows histological and clinical heterogeneity, the studies suggest that the majority of PDA expresses a successive accumulation of highly penetrant genetic changes at genetic genes such as K-ras, p53, CDKN2A and smad4/DPC4^6^ as well as epigenetic alterations^1^. In endocrine origin, pancreatic neuroendocrine neoplasms (PENs) account for 5% of all pancreatic malignancies which include both cystic and solid PENs^7^. Cystic PENs is estimated to account for up to 11.5% of all PENs^8^,^9^, cystic PENs had less aggressive behavior compared to the solid PENs^7^. Cystic PENs are thought to be developed from solid counterparts as a result of degeneration, necrosis and hemorrhage of tumors^10^.

Tumor suppressor CDKN2A gene is located on chromosome 9p21, which is one of the crucial defenses against cancer development. A large body of evidence suggests that CDKN2A is a target of inactivation in pancreatic cancer^11^. In addition to homozygous deletions and mutation, frequent 5′-CpG island methylation of CDKN2A gene promoter resulting in transcriptional silencing of this gene is noted as an important event in the development of pancreatic cancer. Improved understanding of the role of CDKN2A in pancreatic cancer may offer a tool to refine diagnosis and therapeutic management of pancreatic cancer patients.

The aim of this study was to review the available publications and to summarize the data using meta-analysis to characterize the clinical significance of CDKN2A gene promoter methylation in the pancreatic tumorigenesis.

## Results

### Identification of relevant studies

**Figure 1:**
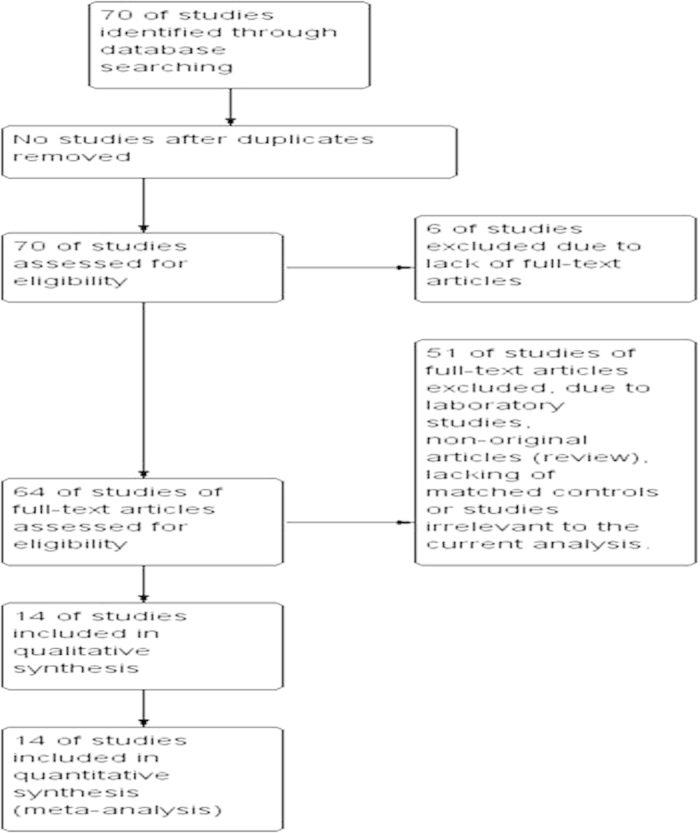
Flow chart of study selection.

Seventy publications were identified by the search method as described above. Fifty-six of those were excluded due to laboratory studies, non-original articles (review), lacking of matched controls or studies irrelevant to the current analysis. Eventually, there were 14 studies included in final meta-analysis (Fig. 1).

### Study characteristics

Fourteen studies published from 2002 to 2012 were eligible for meta-analysis^12^,^13^,^14^,^15^,^16^,^17^,^18^,^19^,^20^,^21^,^22^,^23^,^24^,^25^. A total of 418 pancreatic cancer, 155 PanINs and 45 chronic pancreatitis (CP) patients from China, Singapore, Japan, Germany, England, and United States (USA) were enrolled. Their basic characteristics were summarized in Table 1.

### CDKN2A methylation and clinicopathological features

#### The inactivation of *CDKN2A* through methylation in chronic pancreatitis (CP)

**Figure 2:**
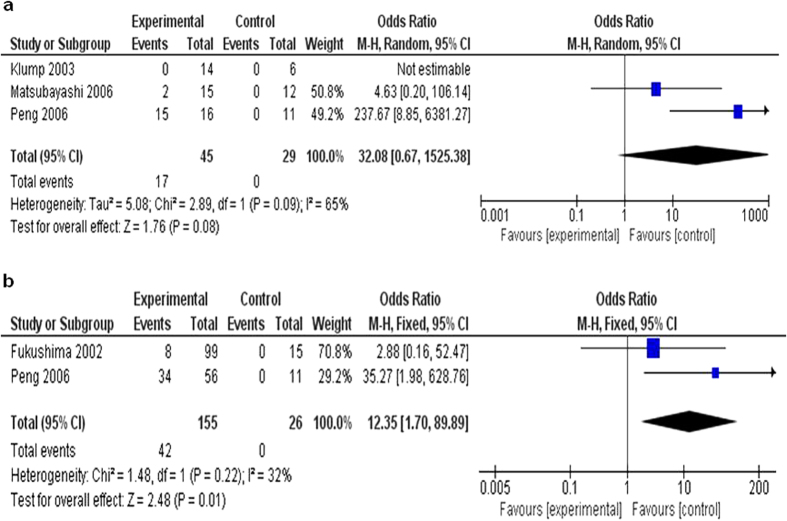
The studies included to investigate *CDKN2A* methylation status between (**a**) 45 chronic pancreatitis patients and 29 normal individuals with the combined OR being 32.08 (95% CI: 0.67–1525.38; Z = 1.76; p = 0.08), and (**b**) 155 PanINs patients and 26 normal healthy controls with the pooled OR being 12.35 (95% CI: 1.70–89.89; Z = 2.48; p = 0.01).

We observed that frequency of *CDKN2A* methylation was higher in chronic pancreatitis than that in normal individual controls, but it did not reach significant difference. The pooled OR from 3 studies including 45 patients with chronic pancreatitis and 29 healthy individuals is shown in Fig. 2a (OR = 32.08, 95% CI = 0.67–1525.38, P = 0.08). These findings indicate that although an increased risk was identified in CP patients for the development of pancreatic cancer, *CDKN2A* gene methylation is not the only determinant for its malignancy. Another study showed that *CDKN2A* methylation was detected in 10% of CP. We excluded the article because no healthy individual controls were available^26^.

#### The inactivation of *CDKN2A* through methylation in pancreatic intraepithelial neoplasia (PanINs)

We observed that the frequency of *CDKN2A* methylation was significantly higher in PanINs patients than that in normal individual controls. The pooled OR from 2 studies including 155 patients with PanINs and 26 healthy individuals is shown in Fig. 2b (OR = 12.35, 95% CI = 1.70–89.89, P = 0.01). PanIN is considered the precursor lesion of invasive pancreatic cancer^27^. Our findings indicate that *CDKN2A* gene methylation could be one of the determinants for its malignancy.

#### The inactivation of *CDKN2A* through methylation in pancreatic cancer

**Figure 3:**
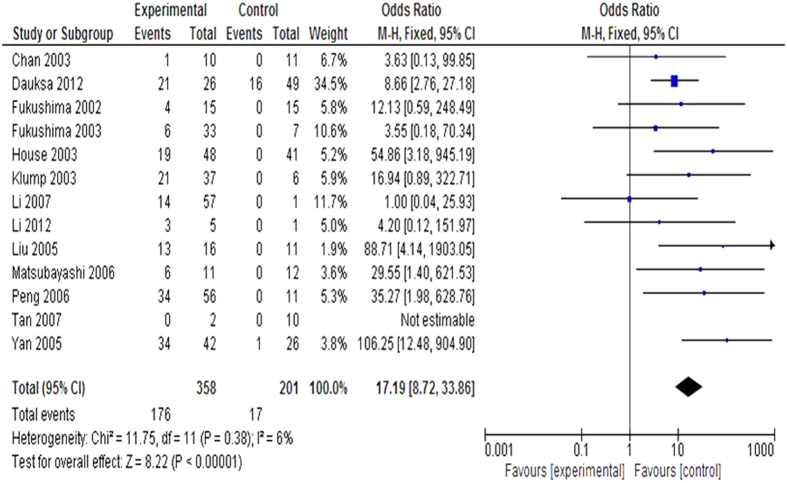
The studies included to investigate *CDKN2A* methylation status between 358 patients with pancreatic cancer and 201 normal individuals. The combined OR was 17.19 (95% CI: 8.72–33.86; Z = 5.26; p < 0.00001).

We observed that the frequency of *CDKN2A* methylation was significantly higher in pancreatic cancer patients than in normal healthy controls. The pooled OR from 13 studies including 358 pancreatic cancer patients and 201 normal individuals is shown in Fig. 3 (OR = 17.19, 95% CI = 8.72–33.86, P < 0.00001), indicating that *CDKN2A* inactivation through methylation plays an important role in the pathogenesis of pancreatic cancer.

**Figure 4:**
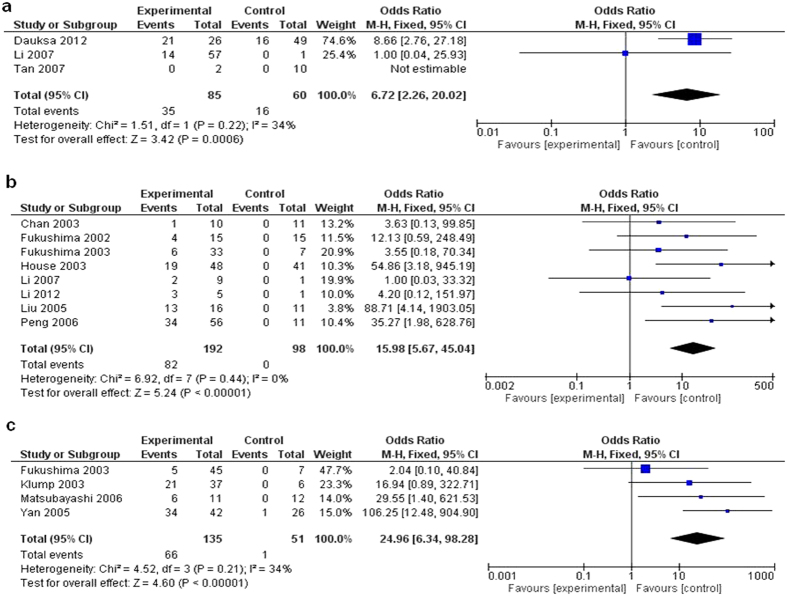
Pooled results of methylation analysis of *CDKN2A* gene in different samples in pancreatic cancer. The pooled OR for blood analysis is shown in Fig. 4a (OR = 6.72, 95% CI = 2.26–20.02, P = 0.0006). As shown in Fig. 4b,c, the pooled OR for pancreatic tissue is 15.98 (95% CI = 5.67–45.04, Z = 5.24, P < 0.00001) and for pancreatic juice is 24.96 (95% CI = 6.34–98.28, Z = 4.60, P < 0.00001).

Previously, diagnostic accuracy of some biomarkers such as K-ras analysis for pancreatic carcinoma has been shown to be diverse in different samples;^28^ therefore, we stratified the analysis of *CDKN2A* methylation by specimen types (blood, pancreatic tissue and pancreatic juice) for pancreatic carcinoma. The pooled OR for blood analysis from 3 studies including 85 pancreatic cancer patients and 60 healthy controls is shown in Fig. 4a (OR = 6.72, 95% CI = 2.26–20.02, P = 0.0006). We further calculated the sensitivity, specificity of *CDKN2A* methylation and other parameters in blood samples from pancreatic cancer patients as described in published literature^29^. Based on Table 2 which is derived from Fig. 4a the results are as follows: sensitivity is 41.0% (a/a + c = 0.41/0.41 + 0.59), specificity is 73.0% (d/d + b = 0.73/0.27 + 0.73), positive predictive value (PPV) is 57.6% (a/a + b = 0.41/0.41 + 0.27), and negative predictive value (NPV) is 57.6% (0.73/0.59 + 0.73).

As shown in Fig. 4b,c, the pooled OR for pancreatic tissues from 8 studies having 192 pancreatic cancer patients and 98 healthy controls was 15.98 (95% CI = 5.67–45.04, Z = 5.24, P < 0.00001) and for pancreatic juice from 4 studies including 135 pancreatic cancer patients and 51 normal individual controls was 24.96 (95% CI = 6.34–98.28, Z = 4.60, P < 0.00001). The overall methylation frequency of *CDKN2A* in blood, pancreatic tissue and juice for pancreatic carcinoma were more than that in normal controls, suggesting a potential role of *CDKN2A* methylation analysis in diagnosing pancreatic cancer.

**Figure 5:**
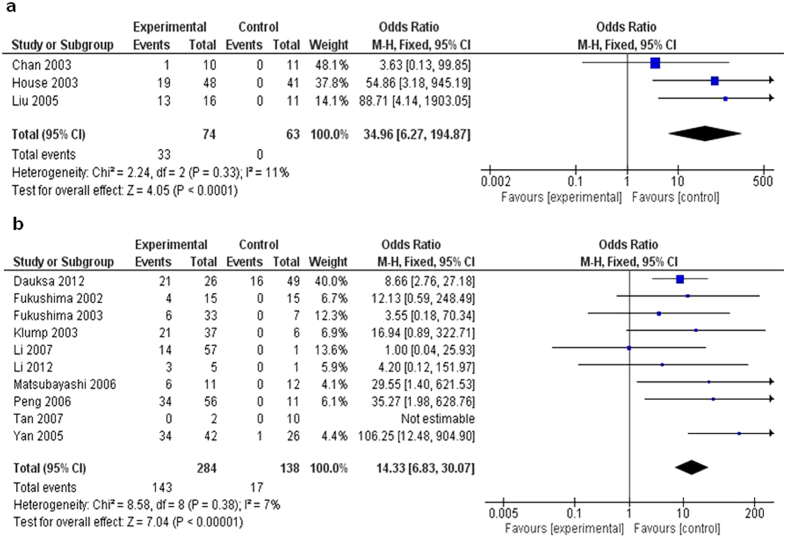
Pooled results of methylation analysis of *CDKN2A* gene PENs and PDA. The pooled OR for PENs analysis is shown in Fig. 5a (OR = 34.96, 95% CI = 6.27–194.87, Z = 4.05, P < 0.0001). As shown in Fig. 5b, the pooled OR for PDA is 14.33 (95% CI = 6.83–30.07, Z = 7.04, P < 0.00001).

PDA remains one of the most deadly malignancies worldwide with extremely poor overall survival^4^. While PENs, an indolent neuroendocrine tumor which may secret neuropeptides causing clinical manifestations, is rare, the estimated incidence of PENs in USA has increased by almost 10 times over the past decades^7^,^11^. The prognosis of PENs cannot be reliably predicted from histopathological assessment because of neuropeptides secretion^19^. This is the justification for us to make meta-analysis of *CDKN2A* methylation in PDA and PENs separately. As shown in Fig. 5a, PENs patients display significantly enhanced *CDKN2A* methylation frequency (OR = 34.96, 95% CI: 6.27–194.87, Z = 4.05, P < 0.0001) compared to controls. As shown in Fig. 5b, the same observation was obtained for PDA patients when compared to corresponding controls (OR = 14.33, 95% CI = 6.83–30.07, Z = 7.04, P < 0.00001). These data indicated that loss of *CDKN2A* gene expression through epigenetic modification correlated with both types of aforementioned pancreatic cancers.

#### Prognostic values of *CDKN2A* gene methylation in PENs/pancreatic cancers (PCs)

**Figure 6:**
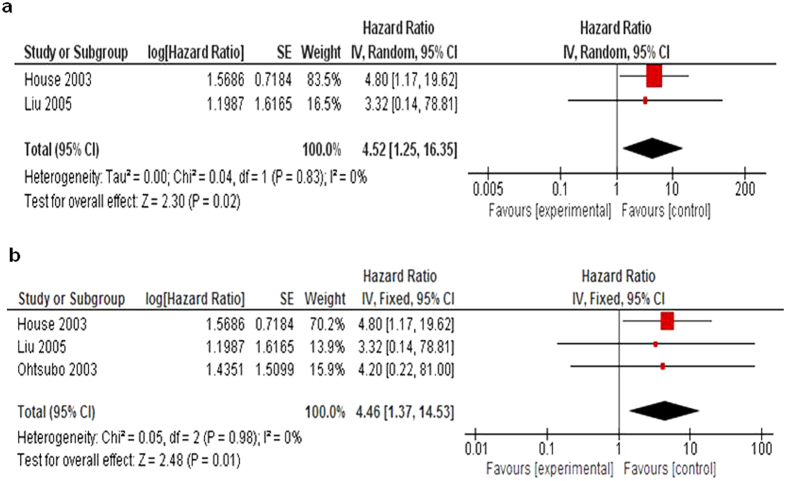
All three included studies estimated the relationship between OS and *CDKN2A* methylation. (**a**).The pooled HR for OS showed that *CDKN2A* hypermethylation was associated with worse survival in PENs, HR = 4.52, 95% CI = 1.25–16.35, P = 0.02. (**b**). The pooled HR for OS showed that *CDKN2A* hypermethylation was associated with worse survival in pancreatic cancer, HR = 4.46, 95% CI = 1.37–14.53, P = 0.01.

Only two included studies^18^,^22^ estimated the relationship between overall survival (OS) in PENs and *CDKN2A* methylation, the pooled results (Fig. 6a) showed the presence of prognostic impact of *CDKN2A* gene methylation on PENs patients (OR = 4.52, 95% CI = 1.25–16.35, Z = 2.30, P = 0.02). The hazard ratio is shown numberically in the fifth column, the confidence interval of the summary of hazard ratio does not include 1.0 (it is 1.25–16.35) suggesting that the association is statistically significant. Ohtsubo *et al.*^24^ reported that the survival period was significantly shorter in patients with pancreatic carcinoma with *CDKN2A* hypermethylation than those with a normal *CDKN2A* gene expression. Combined survival data from all three studies (Fig. 6b) showed OS tended to be shorter in pancreatic cancer patients with epigenetic abnormalities of *CDKN2A* than in PCs with normal expression of *CDKN2A* gene (OR = 4.46, 95% CI = 1.37–14.53, Z = 2.48, P = 0.01). In detail, the hazard ratio is shown numberically in the fifth column, the confidence interval of the summary of hazard ratio does not include 1.0 (it is 1.37–14.53) suggesting that the association is statistically significant. Another study^30^ was excluded from the analysis because only the narrative description can be found, and we were unable to calculate the pooled HR for OS.

#### Association between smoking and *CDKN2A* methylation status

**Figure 7:**
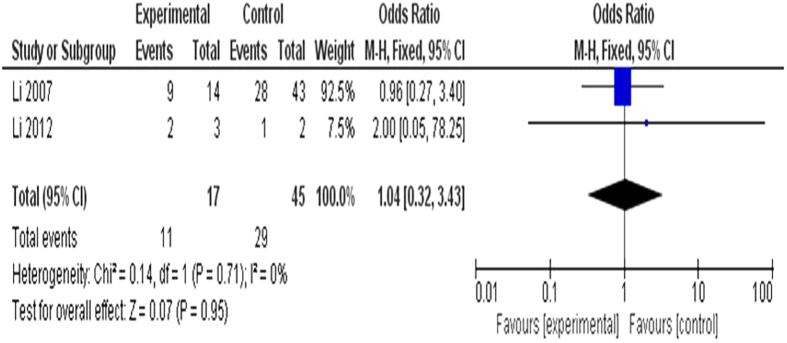
Association between smoking and *CDKN2A* methylation status. The pooled OR from two studies including 17 pancreatic cancer patients and 45 controls is 1.04 (95% CI = 0.32–3.43, Z = 0.07, P = 0.95) which indicated no correlation exists between smoking and hypermethylation status of *CDKN2A* gene.

Cigarette smoking has been considered to increase the risk of pancreatic cancer;^31^ therefore, we evaluated the relationship between methylation of *CDKN2A* and smoking status, in another word, the changes in frequency of *CDKN2A* alterations by smoking status. The pooled OR from two studies including 17 pancreatic cancer patients and 45 controls was 1.04 (95% CI = 0.32–3.43, Z = 0.07, P = 0.95), indicating no correlation between smoking and hypermethylation status of *CDKN2A* gene (Fig. 7).

#### Sensitivity analyses and publication bias

**Figure 8:**
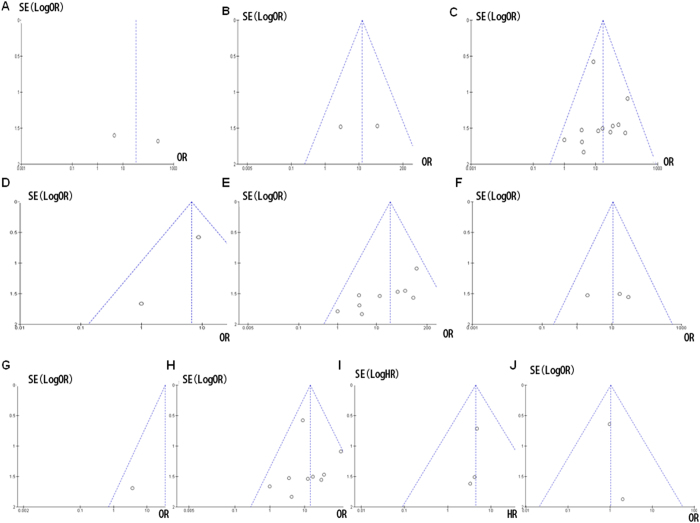
Funnel plot of publication bias in the meta-analysis of *CDKN2A* hypermethylation and clinicalpahological features. *CDKN2A* methylation in CP (**A**), PanINs (**B**), PC (**C**), blood (**D**), pancreatic tissue (**E**), pancreatic juice (**F**), PENs (**G**), PDA (**H**), overall survival (**I**), and smoking **(J**).

A sensitivity analysis, in which one study was removed at a time, was conducted to assess the result stability. The pooled ORs were not significantly changed, indicating the stability of our analyses. The funnel plots were largely symmetric suggesting there were no publication biases in the meta-analysis of *CDKN2A* gene methylation/expression and clinicopathological features (Fig. 8).

## Discussion

Located in the retroperitoneum of individuals who show non-specific symptoms, pancreatic carcinoma is unlikely to be detected until it has reached an advanced stage in most of patients^32^. It has remained one of the most devastating and difficult tumors to diagnose and treat. Due to the absence of disease-specific manifestations, there is an urgent need for reliable biomarkers and new therapeutic target(s) in pancreatic carcinoma. Although the tumor suppressor genes K-ras, p53, CDKN2A and SMAD4 have found to be the central molecular genetic pathways in pancreatic cancer^33^,^34^,^35^,^36^, the gained survival advantage targeting aforementioned pathways remain limited. Other molecular events such as epigenetic changes have recently been identified to contribute to the initiation and progression of pancreatic cancer^37^. In the current study, we concluded that (1) *CDKN2A* inactivation through methylation plays an important role in the pathogenesis of pancreatic cancer (both in PDA and PENs), and could be one of the determinants for its malignancy as supported by higher CDKN2A methylation frequency in premalignant lesion, PanINs than in normal controls, (2) all type of samples such as blood, pancreatic tissue and juice have a potential role for *CDKN2A* methylation analysis in diagnosing pancreatic cancer, (3) overall survival tend to be shorter in pancreatic patients with epigenetic abnormalities of *CDKN2A* than in PCs with normal expression of *CDKN2A* gene, and (4) no correlation exists between smoking and hypermethylation status of *CDKN2A* gene.

As the differential diagnosis between pre-malignant/malignant diseases and normal/benign lesions, the detection of specific tumor markers in blood and pancreatic tissues is a convenient and attractive diagnostic tool. In addition to qualitative analysis, Li *et al.* further quantitatively evaluated methylation levels using the SIRPH (SNuPE combined with ion pair reverse phase HPLC)^38^,^39^ protocol and compared the DNA methylation levels in peripheral blood and cancer tissue for a panel of genes in pancreatic cancer. They found three different groups of methylation patterns. The first group of genes presented higher methylation levels in cancer tissues than in blood DNA (CDKN2A, APC, and DAPK1). In the second group, methylation levels were approximately equal (BCL2, CD44 and TNFRSF10), while in the third group, the DNA methylation levels of ACIN1 were lower in pancreatic tissues than in blood. Overall, methylation alterations in blood could provide a promising approach for early detection of pancreatic cancer^12^. The sensitivity, specificity and other parameters based on Table 2 demonstrated that sensitivity is 41.0% (a/a + c = 0.41/0.41 + 0.59), which indicated that the probability of being test positive when disease present; specificity is 73.0% (d/d + b = 0.73/0.27 + 0.73), which indicated the probability of being test negative when disease absent; positive predictive value (PPV) is 57.6% (a/a + b = 0.41/0.41 + 0.27), which indicated the probability of patient having disease when test is positive; and negative predictive value (NPV) is 57.6% (0.73/0.59 + 0.73), which indicated the probability of patient not having disease when test is negative. More interestingly, the methylation rates detected in pancreatic secretions endoscopically retrieved from the pancreatic duct proved to be somewhat higher than those detected in pancreatic tissues^21^. In parallel, some studies have investigated the alterations of molecular biomarkers other than methylation patterns in pancreatic secretions from pancreatic cancer patients. For instance, K-ras mutations in pancreatic juice of pancreatic cancer patients have been considered as a potential diagnosis tool for pancreatic cancer with acceptable specificity and sensitivity^28^.

Chronic pancreatitis is an important predisposing condition resulting in pancreatic malignancy^40^. The analysis obtained by us displayed that *CDKN2A* hypermethylaion in chronic pancreatitis is higher than those of normal pancreatogram and lower than those of pancreatic carcinoma^21^, suggesting a specific role of CDKN4A in the development of malignant pancreactic diseases, although the difference of CDKN2A methylation frequency between chronic pancreatitis patients and controls in the present study did not reach statistical significance. In another word, CDKN2A changes, especially promoter hypermethylation might imply high-risk precursors in chronic pancreatitis that might develop to cancer^26^. However, larger studies are needed to be carried out to explore the true situation. A study published by Moore *et al.*^41^ has addressed that distinct molecular pathways may be involved in exocrine and endocrine tumorigenesis of the pancreas. In this context, exclusively exocrine pancreatic adenocarcinomas of ductal origin (PDA) and endocrine origin (PENs) have been included, de novo CDKN2A promoter hypermethylation was detected and shown to contribute to the tumorigenesis in both types.

In support of our conclusion that overall survival tends to be shorter in pancreatic patients with epigenetic abnormalities of *CDKN2A* than in PCs with normal expression of *CDKN2A* gene, Gerdes *et al.*^30^ found reduced survival in patients with CDK2A alterations indicating CDKN2A is a prognostic marker in resected ductal pancreatic cancer. This article was excluded from the study because of no matched control.

It needs to be emphasized that the epigenetic alterations other than CDKN2A promoter hypermethylation also contribute to the development of pancreatic cancer. The other well known epigenetic mechanisms are histone modifications [histone deacetylation by histone deacetylases (HDACs) and histone methylation by histone methyltransferases (HMT)] and microRNAs (miRNAs). TGFBR2^42^ and CDH1^43^ are examples which can be regulated by HDACs while EZH2^44^ and SUV39H1^37^,^45^ are regulated by HMT in pancreatic cancers. An increasing number of miRNAs have been shown to be associated with pancreatic tumors^46^,^47^. The most well-known one is miR-21, which is upregulated in pancreatic cancer and targets many apoptosis related genes including PTEN and PDCD4, resulting in inhibited apoptosis and consequently, increased tumorigenicity.

No single technique of DNA methylation detection is appropriate for every application. Some limitations exist in this current analysis. First, DNA methylation at specific loci are dependent on modification of DNA by sodium bisulfate. Although the studies used quantitative gene-specific methylation analysis to link DNA methylation to functional outcomes, bisulfate sequencing is the ideal standard for mapping allele-specific methylation across CpG locations. Without allele-specific measurement, the difference between the status of mosaic methylation of individual alleles or complete methylation of a subpopulation of alleles cannot be distinguished^48^. Second, the current protocol is unable to differentiate 5-methylcytosin (5mc) from 5-hydroxy-cytosine (5hmc)^49^,^50^. Third, similar to gene expression microarrays improving the study of transcriptional regulation, locus-specific DNA methylation on a genome-wide scale will revolutionize and facilitate the DNA methylation analysis^50^. It would be possible to map-out genome-wide DNA methylation patterns from distinct subtype of pancreatic cancer patients. This analysis would result in new insight into the pathogenesis of cancer and open up new avenues of drug discovery and targeted therapies for all kinds of cancer patients.

Taken all together, due to the poor prognosis of pancreatic cancer, understanding the molecular events such as epigenetic changes in pancreatic cancer that drive this aggressive disease is the core for development of early diagnostic tools and more effective therapeutic strategies.

## Conclusion

The results of our study strongly suggest that *CDKN2A* methylation is correlated with an increased risk of pancreatic cancer. *CDKN2A* methylation plays a critical role in pancreatic carcinogenesis and may serve as a prognostic marker.

## Methods

### Search strategy

Medline, Pubmed, Web of Science, Scopus and Embase were searched in August 2014 using the search terms: ‘p16’, ‘p16^INK4a^’, “CDKN2A”, ‘methylation’, ‘pancreatic cancer’, “pancreatic carcinoma” and ‘clinical studies’. Investigations identified through the search approach as described above were screened by titles first, then by abstracts of the publications. After exclusion of non-relevant publications and identifications of duplicates from the different databases, the remaining papers were evaluated in the full text version for in- and exclusion criteria and for relevant articles in the reference lists. All clinical studies except case reports were chosen, for instance, randomized controlled trials (RCTs), cohort studies, case-controls studies and case series. The language of publication was restricted to English. All searched data were retrieved. Authors’ bibliographies and references of selected studies were also searched for other relevant studies. The most complete study was chosen to avoid duplication if same patient populations were reported in several publications.

### Selection criteria

We collected all eligible articles about the relationship between CDKN2A methylation and/or expression and clinicopathological features and clinical outcomes in pancreatic cancer patients for this meta-analysis. Studies meeting the following inclusion criteria were included: (1) CDKN2A methylation and/or expression which were evaluated in the circulation, pancreatic juice, and/or pancreatic tissues, (2) researches which revealed the relationship between CDKN2A methylation and/or expression and pancreatic cancer clinicopathological parameters and prognosis, (3) CDKN2A methylation and/or expression which were examined by methylation specific polymerase chain reaction (MSP), (4) articles which were published as a full papers in English, (5) articles which provided sufficient information to estimate hazard ratio (HR) about overall survival (OS) and 95% confidence interval (CI) and probabilities for overall survival (OS) where applicable. The exclusion criteria included the following: (1) letters, reviews, case reports, conference abstracts, editorials, expert opinion, and non-English language papers; (2) articles having no information on OS or those that could not calculate the HR about OS from the given information; and (3) all publications regarding *in vitro/ex vivo* studies, cell lines and human xenografts were also excluded.

### Data extraction

Two investigators independently extracted data from eligible studies. Disagreements were resolved by discussion till consensus were achieved. Two investigators reviewed all of the articles that fit inclusion and exclusion criteria. The following information was recorded for each study: the name of first author, year of publication, sample source, number of cases, clinicopathological parameters, stage, CDKN2A methylation and/or expression, and patient survival. Data for study characteristics and clinical response were summarized and the data were turned into table format. Heterogeneity of investigation was evaluated to determine whether or not the data of the various studies could be analyzed in a meta-analysis.

### Statistical analysis

Analysis was conducted using the Stata 12.0 (Stata Corporation,TX, USA) and Review Manager 5.2 (Cochrane Collaboration, Oxford, UK). Comparisons of dichotomous measures were done by pooled estimates of odds ratios (ORs) as well as their 95% CIs. P value of <0.05 was considered to be statistically significant. Heterogeneity was examined by a chi-square test with significance being set at P < 0.10; the total variation among studies was estimated by I square. We used I square statistic to assess heterogeneity. The I square value is an estimate of variance due to between-study heterogeneity rather than chance (the Cochran Q statistics). Substantial heterogeneity exists when I square exceeding 50%. If there was heterogeneity among studies, we used a random effect model to pool the ORs; otherwise, a fixed effect model was selected.

The database search generated 70 articles from Medline, Pubmed, the Web of Science, Scopus and Embase. After initial screening of all titles, abstracts and eligibility, 14 full-text studies were retrieved for a more detailed assessment. The search of the article references did not produce additional publications. Eventually, 14 publications met the inclusion criteria for qualitative study and meta-analysis. The article search and study selection are depicted in Fig. 1.

## Additional Information

**How to cite this article**: Tang, B. *et al.* Clinicopathological Significance of *CDKN2A* Promoter Hypermethylation Frequency with Pancreatic Cancer. *Sci. Rep.* **5**, 13563; doi: 10.1038/srep13563 (2015).

## Figures and Tables

**Table 1:** Basic characteristics of the included studies.

Study/Country	Patients/samples	Methods	Primary Aim	Methylation site
Dauksa 2012 12/Germany	26/blood	MSP, SIRPH	Study whether methylation changes in blood could provide a method for early detection of PDA	Promoter, CpG islands
Li 2012^13^/China	5/tissue	MSP	Investigate whether epigenetic modification via hypermethylation represents a mechanism for the inactivation of CDKN2A gene in PDA	Promoter, CpG islands
Tan 2007 14/Singapore	2/blood	MSP	Detect whether serum methylation of CDKN2A is a marker for PDA	Promoter, CpG islands
Li 2007^15^/USA	57/blood, 9/tissue	MSP	Detect whether plasma DNA might be a useful surrogate in epigenetic alterations of PDA	Promoter, CpG islands
Matsubayashi 2006^16^/USA	11/PJ	MSP	Examine whether aberrantly methylated DNA in PJ is an approach for diagnosis of PDA	Promoter, CpG islands
Peng 2006^17^/Japan	56/tissue	MSP	Study whether accumulation of DNA methylation of multiple tumor-related genes is involved in multistage carcinogenesis of pancreas	Promoter, CpG islands
Liu 2005^18^/USA	16/tissue	MSP	Detect whether epigenetic changes in PENs vary in age, histopathologic type and metastasis	Promoter, CpG islands
Chan 2003^19^/USA	10/tissue	MSP	Determine whether methylation profile of PENs differs from carcinoid tumors	Promoter, CpG islands
Yan 2005^20^/England	42/PJ	MSP	Utilize molecular analysis to detect PDA in high-risk groups	Promoter, CpG islands
Klump 2003^21^/Germany	37/PJ	MSP	Determine a role for CDNK2A as a diagnostic marker in differentiation of benign and malignant pancreatic disease	Promoter, CpG islands
House 2003^22^/USA	48/tissue	MSP	Study whether methylation of TSG was an independent predictor of early PENs recurrence and OS following surgical resection	Promoter, CpG islands
Ohtsubo 2003^24^/Japan	60/tissue	MSP	Detest expression of CDKN2A protein and the clinicopathological parameters	Promoter, CpG islands
Fukushima 2003^25^/USA	33/tissue, 45/PJ	MSP	Examine whether CDKN2A in PJ can be a diagnostic approach for PDA	Promoter, CpG islands
Fukushima 2002^23^/USA	15/tissue	MSP	Examine whether CDKN2A can be an indicator of the potential malignancy of epithelial cells of the pancreas	Promoter, CpG islands

PJ: pancreatic juice, tissue: pancreatic tissue, MSP: methylation-specific PCR, SIRPH: sNuPE with IP-RP-HPLC, PENs: pancreatic neuroendocrine neoplasm, TSG: tumor suppressor gene, OS: overall survival.

**Table 2:** Calculation sensitivity and specificity in blood samples from pancreatic cancer patients.

	**Pancreatic cancer**	**Non-pancreatic cancer**
CDKN2 positive	35/85 = 0.41 (a, TP)	16/60 = 0.27 (b, FP)
CDKN2 negative	50/85 = 0.59 (c, FN)	44/60 = 0.73 (d, TN)

TP: true positive, FP: false positive, FN: false negative, TN: true negative.
